# Optimizing patient recovery: prospective study evaluating compliance and clinical outcomes of enhanced recovery protocols in ovarian cancer following cytoreductive surgery with HIPEC

**DOI:** 10.1515/pp-2024-0017

**Published:** 2025-05-06

**Authors:** S. P. Somashekhar, Kumar C. Rohit, Aaron Fernandes, Vijay Ahuja, Kushal Aggarwal, Esha Shanbhag, K. R. Ashwin

**Affiliations:** Aster International Institute of Oncology Consultant – Surgical Oncologist Aster Hospital CMI, Bangalore, India; Consultant Surgical Oncologist, Aster Whitefield Hospital, Bangalore, India; Department of Surgical Oncology, Aster CMI Hospital, Bangalore, India; Gynecological Oncology, Aster Whitefield Hospital, Bangalore, Karnataka, India; Surgical Oncology, Aster Whitefield Hospital, Bangalore, Karnataka, India

**Keywords:** enhanced recovery after surgery (ERAS), cytoreductive surgery (CRS), hyperthermic intraperitoneal chemotherapy (HIPEC), Epithelial ovarian cancer (EOC), peritoneal surface malignancy (PSM)

## Abstract

**Objectives:**

To evaluate the implementation, compliance, and impact of the enhanced recovery after surgery (ERAS) protocol on perioperative outcomes in patients undergoing cytoreductive surgery (CRS) and hyperthermic intraperitoneal chemotherapy (HIPEC) for Stage IIIc ovarian cancer.

**Methods:**

From September 2020 to March 2022, the ERAS protocol (62 perioperative and special consideration guidelines) was prospectively implemented in 75 patients. Based on compliance rates, patients were divided into three groups: Group A (<70 %, 13 patients), Group B (70 %–80 %, 52 patients), and Group C (>80 %, 10 patients). Compliance rates, length of stay, postoperative complications, and readmission rates were analyzed. Ethical committee approval was obtained.

**Results:**

The cohort’s average compliance was 74.5 %, with group averages of 68.4 %, 74.4 %, and 82.5 % (p<0.001). Tolerance to normal diet (p=0.008), postoperative ileus (p=0.161), and mobilization rates (p<0.001) improved with higher compliance. Higher compliance also led to shorter hospital stays (p=0.008) and ICU stays (p<0.001). Complications like ileus and infections were lowest in Group C. No significant differences were found in re-surgery or mortality.

**Conclusions:**

Implementation of the ERAS protocol in patients undergoing CRS and HIPEC for Stage IIIc ovarian cancer is feasible and associated with improved postoperative outcomes. Higher compliance with ERAS guidelines significantly reduced length of hospital and ICU stay, enhanced early mobilization, and improved tolerance to diet, while also decreasing postoperative complications. Compliance above 80 % is necessary for achieving optimal outcomes and protocol modifications may improve compliance.

## Introduction

Cytoreductive surgery with hyperthermic intraperitoneal chemotherapy (CRS and HIPEC) is an important therapeutic strategy in the treatment of epithelial ovarian cancer (EOC). Randomized trials, retrospective studies and meta analysis has shown survival benefit [1], [2], [3], [4], [5], [6], following which HIPEC has been included as a treatment option in oncological guidelines [7]. Morbidity and mortality concerns are one of the deterrents for advocating CRS and HIPEC by clinicians [8]. The mortality and morbidity rates for CRS and HIPEC is 1–5 % and 10–30 %, respectively [9], [10], [11], [12].

Enhanced recovery after surgery (ERAS) is evidence-based perioperative measures designed to reduce the exaggerated postoperative metabolic and inflammatory response [13], 14]. ERAS challenges the conventional practices and traditional attitudes of clinicians. These multimodal pathways are widely used in various surgical fields for optimizing patient outcomes with reproducible clinical benefits [15], 16] ERAS pathway has shown to benefit patients by decreasing complications, length of stay and costs [17], [18], [19], [20]. It is personnel-intensive, requiring a multidisciplinary team to optimize patients from the initial assessment in outpatient department to the early days after surgery. The team is inclusive of ERAS coordinator, nutritionists, nurses, physiotherapists and doctors, spanning the entire continuum of care from preoperative counselling to return to normal function. Audit of results, assessing adherence and compliance rates is an essential component of the ERAS program [21].

Hubner et al. published the ERAS guidelines specific to CRS and HIPEC with several key elements including preoperative, intraoperative, post operative aspects and special considerations [22], 23]. The mere introduction of an ERAS protocol does not ensure success of program as there exists a wide heterogeneity in real world practice of perioperative patient care. Achieving high degree of compliance is imperative for patient benefit and satisfaction [24], 25]. Clinical studies have shown short and long-term prognoses of patients are closely related to the compliance to protocol [26], [27], [28], [29], [30], [31], [32], [33]. It is well known that minimally invasive surgery allows better outcomes with and without ERAS [34]. But can the same benefit of ERAS be reciprocated in a supra invasive surgery like CRS and HIPEC is not known.

This ERAS protocol represents a significant change in practice for CRS and HIPEC and ours is the first study to get evidence from the actual application of the guidelines in a clinical setting and analyze the course and effect of implementation of the new ERAS protocol for CRS and HIPEC in a PSM center of excellence. Considering the high morbidity and mortality, it is important that we study the effect of these perioperative protocols on outcomes, the barriers to achieve compliance and problems facing successful implementation.

## Materials and methods

**Figure 1:**
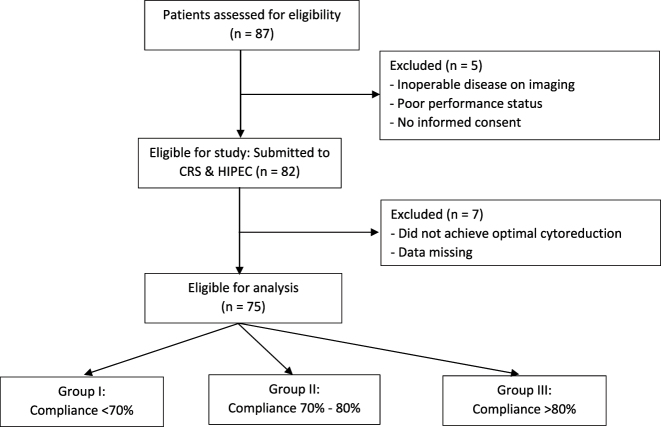
Flowchart of the study participants.

A retrospective analysis of prospectively maintained data at a tertiary referral hospital and centre of excellence for PSM. The centre has ERAS certification with a dedicated ERAS co-ordinator and the protocol is currently part of our routine perioperative care in gynaecological and colorectal surgeries. A total of 87 patients with Stage IIIc ovarian cancer between September 2020 and March 2022 participated in the study. Twelve patients dropped out during the study period, leaving data from 75 patients for outcome analyses. Figure 1 All 75 patients had ERAS protocol applied following informed consent and underwent CRS and HIPEC as per institution protocol [35]. The study was approved by the Institutional Review Board and ethics committee.

Perioperative care was provided in all patients based on the enhanced recovery after surgery society guidelines for perioperative care in CRS and HIPEC, consisting of 52 essential and 10 non-essential components (preoperative, intraoperative and postoperative and special considerations). Ten elements that were defined as non-essential were based on the weak negative or no consensus recommendation in the guidelines [22], 23] Table 1 The team responsible for implementation of the protocol included, 3 surgeons, 2 anaesthetists, 2 nurses, a physiotherapist, a dietician and ERAS coordinator. The ERAS coordinator was responsible for data collection, continuous auditing and monitoring the course of implementation, ensure adherence to the protocol, assess the compliance rates, record variations in daily practice and identify problems encountered during the process. Demographic factors like age, medical comorbidity, upfront surgery or interval debulking surgery pre-, intra-, and postoperative data was collected. Patients were discharged if they met the discharge criteria: no intravenous fluids, no infection, tolerating solid food, adequate pain control with oral analgesics and independent ambulation. Patients were contacted by telephone at 30 and 90 days following surgery.

**Table 1:** ERAS protocol for CRS + HIPEC.

Parametrs	
**PREOPERATIVE**	

ESSENTIAL 1. Preadmission information, education and counselling 2. Preoperative optimisation: Intensive alcohol cessation program Intensive behavioural intervention for smokers Preoperative anemia screening and treatment 3. Physical exercise/prehabilitation 4. Nutritional care: Screening, supplementation Preoperative nutritional screening Nutritional/Protein supplementation 5. Preoperative anaesthetic assessment Assessment of cardiac risk Screening for obstructive sleep apnea Complete laboratory testing Frailty screening 6. Post-operative nausea and vomiting (PONV) Use of antiemetic drugs	7. Pre-anaesthetic medication Preoperative multimodal analgesia 8. Selective preoperative bowel preparation + oral antibiotic Yes for probable rectal resection Oral antibiotic decontamination in all 9. Preoperative fasting and carbohydrate treatment 6 h for solids and 2 h for liquids b.Carbohydrate pre loading NON ESSENTIAL 10. Oral immunonutrition 11. Total-intravenous anaesthesia 12. No preoperative use of sedative/anxiolytics 13. No for probable colectomy

**INTRAOPERATIVE**	

ESSENTIAL 1. Antimicrobial prophylaxis and skin preparation Preoperative antimicrobial prophylaxis Skin preparation by chlorhexidine Antiseptic shower, shaving and adhesive drapes No postoperative antibiotic prophylaxis 2. Standard anaesthetic protocol Cricoid pressure during rapid sequence intubation Epidural analgesia/TAP blocks Multimodal analgesia (low tidal volumes) Routine protective mechanical ventilation Cardiac output monitoring Use of deep neuromuscular antagonists and reversal 3. Intraoperative normothermia Prevention of hypothermia Prevention of hyperthermia	4. Intraoperative normoglycemia 5. Perioperative fluid management Goal directed fluid therapy and catecholamines Use of crystalloids Limiting postoperative fluid-related weight gain 6. Restrictive blood transfusion policy (level of 8 g/dL) 7. Abdominal and thoracic drains Abdominal drains Thoracic drains 8. Early extubation NON ESSENTIAL 9. Tranexemic acid 10. Prothrombin complex concentrate 11. Pre-emptive FFP

**POST OPERATIVE**	

ESSENTIAL 1. Nasogastric drainage 2. Early removal of urinary catheter (postoperative day 3) 3. Prevention of postoperative ileus Postoperative thoracic epidural analgesia/TAP block Postoperative selective m-opioid receptor antagonists Laxatives, prokinetics and adjunct measures 4. Postoperative analgesia Postoperative thoracic epidural analgesia Combination analgesia (paracetamol, NSAIDs and opioids) 5. Perioperative nutritional care Early oral intake (solid food from POD1) Oral nutritional supplements Screening for insufficient intake Pre-emptive parenteral nutrition 6. Postoperative control of glucose 7. Prophylaxis against thromboembolism Mechanical thromboprophylaxis (until complete mobilization) Pharmacological thromboprophylaxis Extended pharmacological thromboprophylaxis	8. Mobilization and exercises Early mobilization: Day of surgery for>2 h and Physical exercises for POD 2 and > 6 h 9. Post-discharge care (nutritional care and physiotherapy) 10. ERAS audit and reporting 11. Prevention, early detection and treatment of HIPEC complication Early stop of anti-angiogenic medications No high-dose of cisplatin Use of sodium thiosulfate No use of intraoperative loop diuretics and dopamine No high-dose of mitomycin C (MMC) No post-operative administration of GCSF (neutopenia) NON-ESSENTIAL 12. No postoperative use of alternative analgesia: Ketamine, gabapentin 13. Preemptive enteral nutrition 14. No prophylactic ureteral stenting

We analysed the degree of implementation of the protocol and compliance to protocol. The compliance rate for each patient was calculated as the number of interventions fulfilled for the 52 essential elements of protocol. Primary outcome of the study was to analyse the effect of the ERAS protocol compliance rate on length of hospital stay (LOS), recovery parameters (diet tolerance, mobilization), complications, mortality and readmission rate. Complication was defined using the Clavien–Dindo classification. Readmission was identified as any patient rehospitalisation within 30 days of surgery after discharge.

Statistical analysis: The results are presented as mean ± standard deviation (SD), median and interquartile range (IQR), when appropriate. Tests were selected depending on the type of the variables. For the qualitative variables the chi-square test was used. In cases of quantitative variables, where no normal distribution was observed, we used the Kruskal-Wallis test. To compare the two groups, when non-normally distributed quantitative variables were present, the U Mann-Whitney test was used. The relationship between the compliance with the protocol and LOS was examined using Pearson’s correlation. Statistical significance is accepted at p<0.05.

## Results

**Figure 2:**
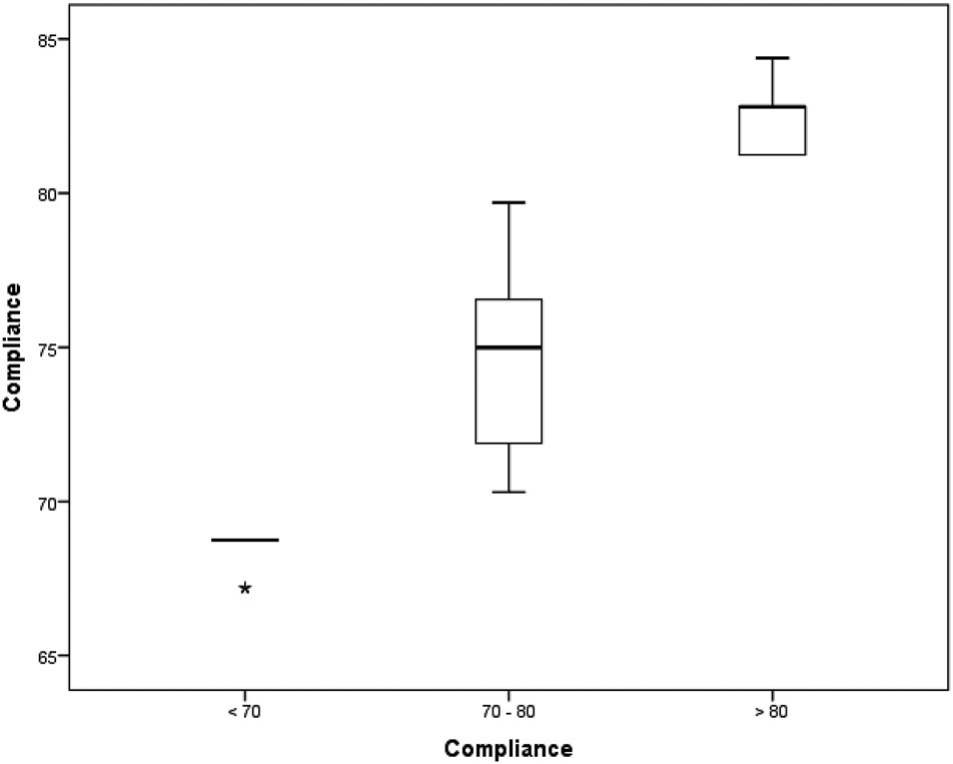
The average compliance in the patient subgroups.

We analysed the compliance with the ERAS protocol and the entire cohort was divided into 3 sub groups according to the compliance score achieved: <70 % (Group A – 13 patients), 70–80 % (Group B – 52 patients) and >80 % (Group C – 10 patients). The average compliance achieved in the study was 74.5 %. The average compliance with the protocol differed significantly between subgroups, 68.4 % in group A, 74.4 % in group B and 82.5 % in group C (p<0.001, Figure 2). Demographic characteristics and operative details in the subgroups were comparable to each other in terms of age, gender, body mass index (BMI), performance score, co-morbidity, peritoneal carcinomatosis index (PCI) and operative times. Table 2.

**Table 2:** Demographic characteristics of compliance sub groups.

Parameter	Compliance group <70 %	Compliance group <70–80 %	Compliance group >80 %	p-Value
No of patients	13	52	10	
Age, mean	56.3 ± 7.36	54.1 ± 9.31	55.7 ± 7.17	0.657
Comorbidity	10 (76.9)	31 (59.6)	4 (40.0)	0.200
Mean ECOG performance score ECOG 0 ECOG 1	8 5	25 27	4 6	0.511
Haemoglobin (mean ± SD)	11.3 ± 1.52	11.1 ± 1.57	11.2 ± 2.08	0.868
Albumin (mean ± SD)	3.9 ± 0.44	3.9 ± 0.41	4.0 ± 0.37	0.861
Mean BMI (kg/m2) (mean ± SD)	23.3 ± 3.88	23.1 ± 3.16	21.7 ± 3.62	0.456
CRS - Upfront - Interval	3 10	17 35	2 8	0.622
PCI (median)	14.0 (2–31)	11.0 (3–39)	9.0 (3–18)	0.669
Mean duration of surgery, hours (Median)	330 (190–380)	370 (230–450)	290 (170–350)	0.470

### Clinical parameters

**Figure 3:**
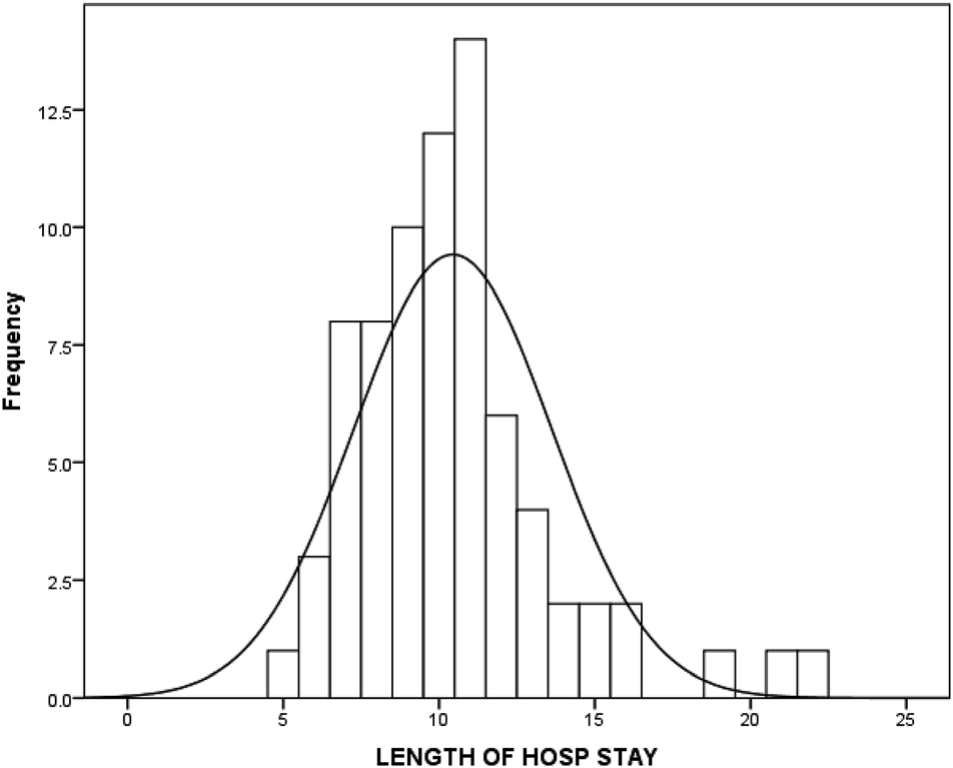
Length of hospital stay in the cohort.

**Figure 4:**
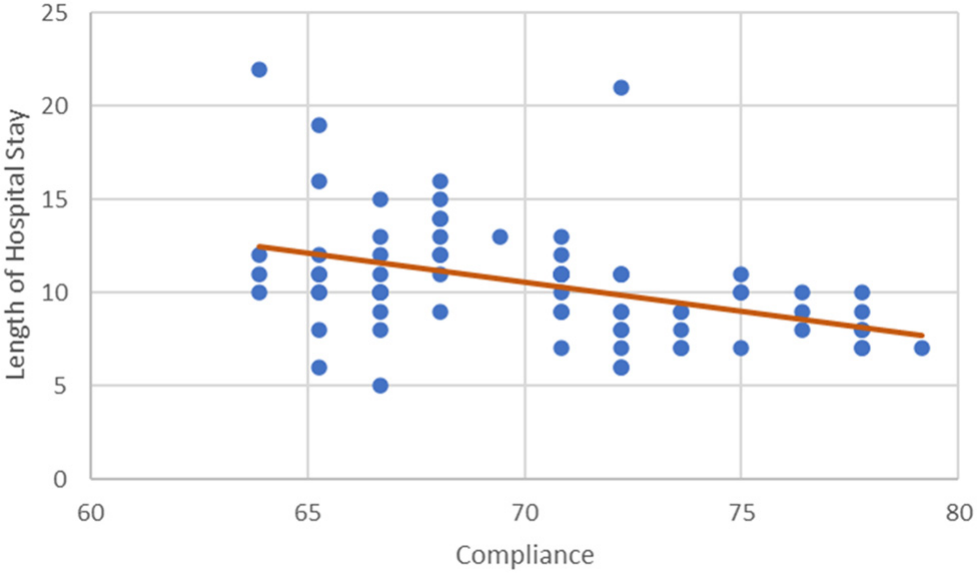
Correlation between compliance and length of hospital stay.

An inverse correlation between compliance LOS and ICU stay was noticed in the study. The LOS was shorter in the higher compliance subgroups; 11.0 (6–22), vs. 10.0 (5–21), vs. 8.0 (7–10) p=0.008) days respectively. Figure 3 and 4 The ICU stay was shorter in better compliant patients; (4.0 (2–8) vs. 2.0 (1–5) vs. 1.0 (0–2) p<0.001), respectively. The on table extubation rate although not statistically significant (p<0.083) did show earlier extubation and avoidance of ICU in higher compliance subgroups. There were no statistically significant differences in postoperative 30-day readmission rate (p<0.438), reoperation rate (p<0.799) and 30-day hospital mortality rate (p<0.438) between the three subgroups. The analysis of recovery parameters showed differences between the subgroups: tolerance of normal diet (10.7 ± 3.42 vs. 8.3 ± 3.03 vs. 5.7 ± 1.42 days; p 0.008), post operative ileus 46.2 % vs. 38.5 % vs. 20 %; p=0.161), mobilization of a patient on the day of surgery (15.4 % vs. 21.2 % vs. 90 % p<0.001). Table 3.

**Table 3:** Comparison of compliance sub groups with clinical parameters.

Parameter	Compliance group<70 %	Compliance group<70–80 %	Compliance group>80 %	p-Value
No of patients	13	52	10	
Length of ICU	4.0 (2–8)	2.0 (1–5)	1.0 (0–2)	0.001
Length of hospital stay, days	11.0 (6–22)	10.0 (5–21)	8.0 (7–10)	0.008
Extubation – on table	16 (61.5 %)	82 (78.8 %)	20 (100.0 %)	0.083
Readmission	1	1	0	0.438
Re surgery	0	1	0	0.799
In hosp mortality	1	1	0	0.438
Tolerance to normal diet, days	10.7 ± 3.42	8.3 ± 3.03	5.7 ± 1.42	0.008
Post operative ileus	6 (46.2 %)	20 (38.5 %)	1 (10 %)	0.161
Mobilization on day of surgery	4 (15.4 %)	22 (21.2 %)	19 (90 %)	< 0.001

### Postoperative complications

Complications were compared in subgroups and was not statistically significant (p=0.849). Grade 1–2 complications were seen in majority of the patients. Eighteen patients (12 %) had grade 3–5 complications; in group A and B (23.1 % and 11.5 % respectively) and none in group C. The most common complications were post operative ileus (36.6 %), surgical site infections (20.6 %), pulmonary complications (17.3 %) and intraabdominal collections (13.3 %). Two patients were readmitted, and both treated conservatively. 0ne patient required reoperation in post operative period because of fecal peritonitis secondary to anastomotic leak. Table 4.

**Table 4:** The incidence of postoperative complications in compliance subgroups.

Calvien Dindo	Compliance						Total		p-Value
	<70		70–80		> 80				
	Count	%	Count	%	Count	%	Count	%	
1	2	15.4	9	17.3	2	20.0	13	17.3	
2	8	61.5	37	71.2	8	80.0	53	70.7	
3	2	15.4	4	7.7	0	0.0	6	8.0	
4	0	0.0	1	1.9	0	0.0	1	1.3	
5	1	7.7	1	1.9	0	0.0	2	2.7	
Total	13	100.0	54	100.0	10	100.0	75	100.0	p=0.849

### Compliance to individual elements of protocol

Compliance to the various modalities varied considerably and was poorest during the postoperative period. Lowest compliance rates between the groups were found for following elements: nasogastric drainage, removal of urinary catheter by post operative day (POD) 3, early oral intake (solid food from POD1), mobilisation on day of surgery>2 h, physical exercises on POD 2 for >6 h, no usage of post operative antibiotic prophylaxis, abdominal and thoracic drains, post-operative fluid overload monitoring and avoidance, which was statistically significant (p<0.001); Routine mechanical bowel preparation and pre-emptive parenteral nutrition showed mild significant difference. Table 5.

**Table 5:** Comparison of ERAS factors with low compliance rate in the compliance sub groups.

Parameter	Recommendation	Compliance group<70 % (n=13)	Compliance group<70–80 % (n=52)	Compliance group>80 % (n=10)	p-Value
Nasogastric drainage	Weak negative	3 (23.1)	35 (67.3)	10 (100.0)	<0.001
Removal of urinary catheter by POD 3	Strong positive	3 (23.1)	39 (75.0)	10 (100.0)	<0.001
Early oral intake (solid food from POD1)	Strong positive	3 (23.1)	35 (67.3)	10 (100.0)	<0.001
Mobilisation: Day of surgery>2 h	Strong positive	2 (15.4)	11 (21.2)	9 (90.0)	<0.001
Physical exercises for POD 2 and >6 h	Strong positive	2 (15.4)	16 (30.8)	9 (90.0)	<0.001
No post operative antibiotic prophylaxis	Weak positive	0 (0.0)	4 (7.7)	9 (90.0)	<0.001
Abdominal and thoracic drains	Weak positive	2 (15.4)	19 (36.5)	10 (100.0)	<0.001
Limiting postoperative weight gain	Strong positive	4 (30.8)	38 (73.1)	10 (100.0)	<0.001
Avoid routine mechanical bowel preparation	Weak positive	12 (92.3)	52 (100.0)	10 (100.0)	0.089
Pre-emptive parenteral nutrition	Strong positive	7 (53.8)	30 (75.0)	10 (100.0)	0.041

## Discussion

Managing advanced stage EOC patients with elderly age, post chemotherapy, previous surgery multiple comorbidities with complex multimodality treatment and significant morbidity represents a clinical challenge. ERAS protocols are designed to offset stress caused by conventional and counter intuitive practices and achieve pretherapy baseline function. There are only few retrospective studies on the use of ERAS protocol in CRS and HIPEC patients till now [36], [37], [38], [39], [40]. It represents a significant change in practice for CRS and HIPEC and poses a challenge for adherence and compliance.

### Impact on clinical outcomes

#### Compliance issues

The dilemma is not only the implementation of ERAS protocols but also ensuring clinical adherence and patient compliance. The impact of different levels of compliance and specific elements is poorly understood. Studies have also shown that the key element for the improvement of recovery and convalescence parameters is to increase compliance [27], 41], 42]. The mean compliance rate varies between 60 % and 80 %, even in centres that use it on a routine basis [43], 44]. In the present study improved compliance resulted in shortened LOS and ICU stay in the higher compliance groups but patients with compliance of less than 70 % failed to achieve benefit. Interestingly a study has also shown that an increase in compliance to the ERAS protocol from>80 % to>90 % was not associated with further improvement in short-term outcomes [32].

It will be difficult to compare our current research with other ERAS studies because of the difference in nature of surgery. It is well known that minimally invasive surgery allows better outcomes compared to open surgery. Research that has demonstrated benefit and safety with ERAS has been with colorectal or gynaecological surgery where minimally invasive surgeries are very common [26], 30], 45]. In a surpra invasive surgery like CRS and HIPEC there is extensive multiquadrant procedures(peritonectomy with multivisceral resections) leading to significant physiological changes and achieving full protocol compliance is impossible in majority of patients [25].

We analysed each individual element of the protocol based on the compliance rate. Elements like prehabilitation, optimization of nutrition, short duration of fasting with carbohydrate loading, multimodal analgesia, postoperative nausea and vomiting (PONV) prophylaxis, anaesthesia induction and ventilation protocols, goal directed fluid therapy, DVT prophylaxis and maintenance of intraoperative normothermia were well adopted with highest compliance rates. There was no statistical significance between the compliance groups for these elements.

We observed deviations from the pathway in certain clinical situations resulting in lower compliance rates for some aspects of protocol, like routine bowel preparation in case of extensive disease in anticipation of multiple resections and anastomosis, prolonged urinary catheter after bladder peritonectomy or resection. In the lowest compliance group, items with lower implementation rates included: nasogastric drainage, removal of urinary catheter by post operative day (POD) 3, early oral intake (solid food from POD1), mobilisation on day of surgery>2 h, physical exercises on POD 2 for>6 h, no usage of post operative antibiotic prophylaxis, abdominal and thoracic drains, post-operative fluid overload monitoring and avoidance. Unfortunately, some clinical situations do not allow full protocol realization due to medical considerations. Although routine NG decompression is not recommended by the protocol, in presence of extensive upper abdominal disease; total supra colonic omentectomy, lesser omentectomy, gastric resections or splenectomy when performed can lead to delayed gastric emptying necessitating post op NG drainage [46]. Guidelines have also recommended against the routine use of peritoneal drains since placement of drains can stimulate serous fluid production, and may lead to an increased risk of surgical-site infection and adhesions without any benefit of early detection of anastomotic leak. After CRS and HIPEC the possibility of intraperitoneal collections is higher and therefore most surgeons prefer placement of intraperitoneal drains after extensive CRS [47], 48]. Aggressive mobilisation and early normal diet initiation is again difficult to comply as most patients who undergo CRS and HIPEC deal with major hemodynamic, respiratory and metabolic derangements needing ICU care with or without ventilatory support [49], 50].

The improvement in recovery outcomes is not an effect of one particular element, but rather an aggregation of gains from all the elements of guidelines. Even though it is not always possible to fully adhere to the protocol, as a whole they are proven to work, which is clearly confirmed in our analysis.

#### Difficulties in ERAS implementation

The full implementation of the comprehensive ERAS guidelines into daily practice for CRS and HIPEC meets certain difficulties. Despite the existence of strong evidence of benefit in surgical practice, introducing a multielement protocol represents a shift from the ingrained conventional practices and its adoption with systematic implementation by HIPEC surgeons will be gradual [51]. Effective implementation of ERAS requires counselling of patients, education and close collaboration of a multidisciplinary team consisting of surgeons, anaesthetists, intensive care specialists, nurses, physiotherapists and dieticians [52], [53], [54].

The support by the hospital administrators and recommendation of guidelines by scientific societies are also significant for success of the program [55].

#### Recommendations

Seventy-two items in the protocol should be stratified as considered as ERAS core items, those with highest level of evidence and with maximum impact on patient outcomes. Peri-operative care items without a specific focus on enhanced recovery could be removed from the guidelines to make them shorter, more specific, and easier to implement. Modifying few elements of protocol depending on clinical requirements and centre capabilities will make ERAS more practical and easier to implement.

There were few limitations in the study. We did not analyze cost effect of ERAS in our study. This was a single-center observational study, multicenter and large-scale trials are needed to verify the current results.

## Conclusions

Compliance at the level of 80 % or more is needed to achieve patient benefit, faster and safer patient recovery with early resumption of adjuvant therapies and ultimately improved quality of life and patient satisfaction. Training and formation of a dedicated team is imperative for success. Although there is evidence of benefit in other specialties where minimally invasive surgery is a major component, the reciprocation of same benefit in highly invasive surgery like CRS and HIPEC requires conclusive evidence to legitimise the use. There is room to improve the protocol and standardize it to achieve higher adherence and compliance.
